# Construction of Loose Positively Charged NF Membrane by Layer-by-Layer Grafting of Polyphenol and Polyethyleneimine on the PES/Fe Substrate for Dye/Salt Separation

**DOI:** 10.3390/membranes11090699

**Published:** 2021-09-13

**Authors:** Shuai Liu, Xiaofeng Fang, Mengmeng Lou, Yihan Qi, Ruo Li, Gang Chen, Yonglian Li, Yanbiao Liu, Fang Li

**Affiliations:** 1 Textile Pollution Controlling Engineering Centre of Ministry of Ecology and Environment, College of Environmental Science and Engineering, Donghua University, Shanghai 201620, China; liushuai5274@163.com (S.L.); mengmeng_lou@outlook.com (M.L.); Yihan525@163.com (Y.Q.); 2202100@mail.dhu.edu.cn (R.L.); cheng@dhu.edu.cn (G.C.); yanbiaoliu@dhu.edu.cn (Y.L.); 2 Shanghai Institute of Pollution Control and Ecological Security, Shanghai 200092, China; 3 Tus-Water (Shanghai) Co., Ltd., Shanghai 200333, China; ylLi123@163.com

**Keywords:** loose nanofiltration, dye desalination, polyphenol, polyethyleneimine, grafting

## Abstract

The effective separation of dyes and inorganic salts is highly desirable for recycling inorganic salts and water resource in printing and dyeing wastewater treatment. In this work, tannic acid (TA) and polyethyleneimine (PEI) were grafted on the PES/Fe ultrafiltration membrane via the coordination assembly and Michael addition strategy to fabricated a loose nanofiltration membrane (LNM). The effect of PEI concentration on membrane morphologies and properties was systematically investigated. The membrane surface becomes more hydrophilic and transforms into positive charge after the PEI grafting. The optimized PES/Fe-TA-PEI membrane possesses high pure water flux (124.6 L·m^−2^·h^−1^) and excellent dye rejections (98.5%, 99.8%, 98.4%, and 86.4% for Congo red, Eriochrome black T, Alcian blue 8GX, and Bromophenol blue, respectively) under 2 bar operation pressure. Meanwhile, the LNM showed a high Alcian blue 8GX rejection (>98.4%) and low NaCl rejection (<5.3%) for the dye/salt mixed solutions separation. Moreover, the PES/Fe-TA-PEI LNM exhibited good antifouling performance and long-term performance stability. These results reveal that such LNM shows great potential for effective fractionation of dyes and salts and recycling of textile wastewater.

## 1. Introduction

With the rapid development of global industrialization, the water consumption and wastewater generation increase annually, which causes serious water shortage and environmental pollution [1,2]. In particular, dyeing wastewater has become a serious environmental problem due to its deep chromaticity, large water quality changes, complex composition, and great harm [3,4]. In the process of dye synthesis, the introduction of inorganic salts to purify the dye products results in a large amount of salt remaining in the dye, which inevitably produces salt-containing dye wastewater [5,6]. In addition, in order to improve the dye uptake rate of the fabric, a large amount of inorganic salt is added during the dyeing process. These inorganic salts give the dye wastewater a high recycling value [7,8]. Therefore, it is very important to realize the effective separation of dye and salt molecules in dyeing wastewater.

The main methods of treating dyeing wastewater include the oxidation method [9], adsorption method [10], and flocculation method [11]. Although these traditional methods are efficient, the inorganic salt is hardly separated from the mixture wastewater and secondary pollution is easily caused with addition of chemicals [12]. Compared with traditional technologies, membrane separation technology exhibits its advantages such as low energy consumption, small spatial requirements, and no secondary pollution in wastewater treatment. Among them, nanofiltration (NF), addressing pore sizes in the range of 0.5–2 nm, can effectively separate monovalent/multivalent ions and retain organic dye molecules, which have been widely used in dyeing wastewater treatment [13,14]. However, traditional commercial NF membranes contain a dense separation layer that have high rejection rates for high-valent salts and dye molecules, and low permeation flux [15,16,17]. The selective separation of dyes and inorganic salts was difficult to achieve. Recently, the loose nanofiltration membrane (LNM) with a relatively large pore size, which facilitates the effective penetration of salt while maintaining a high retention rate of dye molecules, has been extensively researched in dye/salt separation [18,19].

In recent years, polyphenols have been widely used in the preparation of LNM [20,21,22]. As a major representative, dopamine (DA) can self-polymerize in the air to form a polydopamine (PDA) coating, which has been widely used in the preparation of LNM [23,24]. For instance, Wang et al. [25] used CuSO_4_/H_2_O_2_ as the initiator to co-deposit PDA and PEI on the PAN base membrane. The prepared loose nanofiltration membrane had a rejection rate of more than 98% for various dyes and a salt rejection rate less than 5% (Na_2_SO_4_ and NaCl). However, due to the high cost of DA, it is difficult to use it for mass production. Tannic acid (TA), a natural plant polyphenol, is a relative cheaper polyphenolic compound and has abundant phenolic hydroxyl groups [26]. TA can chelate with metal ions to form stable metal/polyphonic network and react with amino compound through Schiff base or Michael addition reactions, which have engaged lots of attention for membrane fabrication [27,28,29,30]. Fan et al. [31] used the coordination reaction of TA and Fe^3+^ to form a TA–Fe coating on the surface of the PES base film. The composite NF membrane had high dye rejection (>90%) and excellent antioxidant property and long-term stability. Chen et al. [32] co-deposited TA, polyethyleneimine (PEI), and halloysite nanotubes (HNTs) on the surface of polyvinylidene fluoride (PVDF) membranes to prepare a novel membrane by surface modification. The results found that the hydrophilicity of membrane was improved. The pure water flux reached 45.1 L·m^−2^·h^−1^, and the rejection of direct blue was as high as 96%. Li et al. [33] used TA and PEI to co-deposit on the membrane surface to fabricate LNM and the membrane showed high permeability (40.6 LMH·bar^−1^) and high dye rejection (99.8% for Congo red) as well as low salt rejection (6.1% for NaCl). At present, most LNMs with TA modification were prepared via co-deposition method. However, since the co-deposition process react quickly, the thickness and pore size of separation layer is difficult to precisely control. In addition, the co-deposited separation layer has weak interaction with the based membrane and the stability is a matter of concern. Therefore, the fabrication of stable separation layer with controllable NF performance is highly critical and desirable.

**Figure 1:**
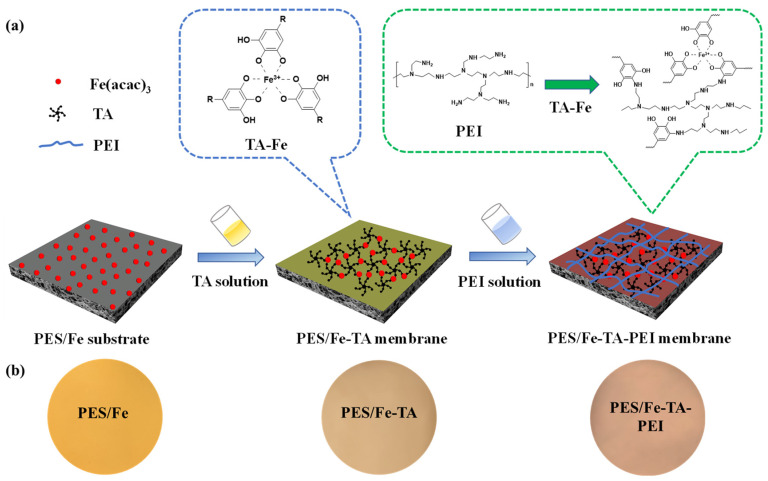
(**a**) Schematic diagram of preparing the PES/Fe-TA-PEI membranes and (**b**) digital pictures of the PES/Fe, PES/Fe-TA, and PES/Fe-TA-PEI membranes.

In this study, an efficient and facile strategy was developed to prepare PES/Fe-TA-PEI LNM via a layer-by-layer grafting strategy. First, TA was grafted on the iron complexes blended PES ultrafiltration membrane by the coordination with Fe^3+^. Then, the TA-PEI composite layer was formed via Michael addition/Schiff base reaction between catechol structures and amino radicals. The preparation process of the LNM is shown in Figure 1a. The thickness of the separation layer was tailored via varying the PEI concentration. The morphology, surface charge, permeability and dye/salt separation performance were investigated in detail. The antifouling performance of prepared membranes was determined using Congo red, Alcian blue 8GX, and Humic acid as model pollutants. Furthermore, the long-term stability in the dye/salt mixed solution was evaluated. The aim of this study is to provide a precise control method to fabricate LNMs for effective separation of dye and inorganic salt in dyeing wastewater.

## 2. Materials and Methods

### 2.1. Chemicals and Materials

Tannic acid (TA), iron (III) acetylacetonate (Fe(acac)_3_), and polyethyleneimine (PEI, average Mw of 70,000, 50 wt%, aqueous solution) were purchased from Aladdin Reagent Co. Ltd. (Shanghai, China). Polyethersulfone (PES, Ultrason E6020P, M_w_ = 58 kDa) was obtained from BASF (Germany) BASF Co., Ltd. (Shanghai, China).and dried at 110 °C for 12 h before use. Polyvinylpyrrolidone (PVP) and N, N-dimethyl formamide (DMF), Congo Red (CR), Eriochrome Black T (EBT), Alcian Blue 8GX (AB 8GX), Bromophenol Blue (BPB), Humic acid (HA), magnesium chloride (MgCl_2_), magnesium sulfate (MgSO_4_), sodium sulfate (Na_2_SO_4_), and sodium chloride (NaCl) were purchased from Sinopharm Chemical Reagent Co., Ltd. (Shanghai, China).

### 2.2. Preparation of the PES/Fe Membranes

The PES/Fe membranes were prepared via nonsolvent induced phase separation (NIPS) method. In detail, PES (16%), PVP (8%), and an Fe(acac)_3_ (1.5%) were dispersed in DMF (78.8%) solution and stirred at 60 °C for 6 h to obtain a uniform casting solution. The PVP was used as a pore-forming agent. The casting solutions were stored at room temperature for 12 h to ensure a complete release of bubbles and then cast on a non-woven support using an automated film applicator with a gap of 320 μm. Subsequently, the cast films were immersed into a coagulation bath at room temperature after being exposed to the atmosphere for 20 s. Then the prepared membranes were immersed in pure water for at least 24 h to leach out the residual solvent before testing.

### 2.3. Preparation of the PES/Fe-TA-PEI Membranes

Firstly, the PES/Fe membranes were immersed in TA solution (10 g/L) and oscillated for 2 h at 35 ℃ with a shaker. The PES/Fe-TA membranes were then thoroughly washed with deionized water to remove the unreacted TA. Then, the PES/Fe-TA membranes were immersed in PEI aqueous solutions with different concentrations (0.5, 1.0, 1.5, and 2.0 g/L) and oscillated for 30 min at 35 °C to obtain the PES/Fe-TA-PEI membranes. Finally, the obtained membrane was stored in deionized water before use.

### 2.4. Characterization of Membranes

The chemical structures and elemental compositions of the membranes were analyzed by Fourier transform infrared spectroscopy (ATR-FTIR, Nicolet 6700, TMO, Waltham, MA, USA) and X-ray photoelectron spectroscopy (XPS, Thermo Fisher Scientific Escalab 250 Xi, Waltham, MA, USA), respectively. The field emission scanning electron microscope (FESEM, Hitachi S-4800, Tokyo, Japan) was operated to characterize the morphology of the membranes. The hydrophilicity of these membranes was characterized by a contact angle goniometer (SL-200C, KINO, Boston, MA, USA) using DI-H_2_O as a probe liquid. The surface zeta potentials of membranes were determined by a Sur-PASS electrokinetic analyzer (SurPASS, Anton Paar GmbH, Graz, Austria).

### 2.5. Performance of Nanofiltration Membranes

The NF experiments were tested by a self-made cross-flow flat membrane module with an effective area of 7.065 cm^2^ at room temperature. The membranes were initially compacted for 20 min under 3 bar to obtain a steady permeation and then the pressure was lowered to 2 bar. The water flux (J, L m^−2^·h^−1^) was measured and calculated by the following equation:

(1) $$J=\frac{V}{A\;\times \;\Delta t}$$

where V (L) is the volume of permeated water, A (m^2^) is the effective membrane area, and Δt (h) is the permeation time.

The rejection performance of these NF membranes was conducted by using single-component salt solution (1 g/L Na_2_SO_4_, MgSO_4_, MgCl_2_ and NaCl) and dye solution (0.1 g/L CR, EBT, AB 8GX, and BPB) as feed solution, respectively. Furthermore, the separation performance of PES/ Fe-TA-PEI composite membrane for dyeing salt mixed system was investigated with different dyes and NaCl solutions. The concentration of fixed dye was 0.1 g/L, the concentration of salt solution was 1 g/L, and the operating pressure was 2 bar. The volume of the feed solution used to perform the filtration experiments was 2 L, and the cross-flow filtration system was operated in recirculation mode. The rejection ratio (R) was defined as the following equation:

(2) $$R=\left(\frac{C_{f}-C_{p}}{C_{f}}\right)\times 100\%$$

where C_p_ and C_f_ was the concentration of permeate and feed solution, respectively. Herein, the salt concentration was measured by an electrical conductivity meter (FE38, METTLER TOLEDO, Shanghai, China). The dye concentration was measured by a UV–vis spectrophotometer (UV-7504PC, XINMAO, Shanghai, China).

### 2.6. Antifouling Performance Measurement and Long-Term Stability Test

The antifouling measurements of the PES/ Fe-TA-PEI membranes were conducted using CR, AB 8GX, and HA as a representative pollutant. The foulant concentrations in feed solutions were 0.1 g/L. The antifouling testing was conducted under 2 bar and the process is as follows: Firstly, the membrane sample was pressurized to reach a stable water flux before the measurement. Then the pure water flux was continuously measured for 60 min and recorded every 10 min as J_w1_. After wards, the membrane filtration was conducted using CR solution as feed for another 60 min. The permeate flux was also tested every 10 min and recorded as J_p_. Later, the membrane after filtering CR solution was simply cleaned by deionized water for 30 min. Subsequently, the pure water flux was measured again and recoded as J_w2_ for 60 min. The operations for AB 8GX and HA antifouling were similar with the above process using AB 8GX or HA solution as the feed solution, respectively. Lastly, the antifouling performance was evaluated by flux recovery ratio (FRR), total fouling ratio (Rt), reversible fouling ratio (Rr), and irreversible fouling ratio (Rir). Those parameters were defined and calculated as follows:

(3) $$\mathrm{FRR}=\frac{J_{w2}}{J_{w1}}\times 100\%$$

(4) $$\mathrm{Rt}=\left(\frac{J_{w1}-J_{p}}{J_{w1}}\right)\times 100\%$$

(5) $$\mathrm{Rr}=\left(\frac{J_{w2}-J_{p}}{J_{w1}}\right)\times 100\%$$

(6) $$\mathrm{Rir}=\left(\frac{J_{w1}-J_{w2}}{J_{w1}}\right)\times 100\%$$

The AB 8GX (0.1 g/L) solutions mixed with NaCl (1 g/L) were used as feed solution to conduct the long-term stability measurement (24 h) on the optimized the PES/ Fe-TA-PEI membrane.

## 3. Results and Discussion

### 3.1. The Physicochemical Characterization

**Figure 2:**
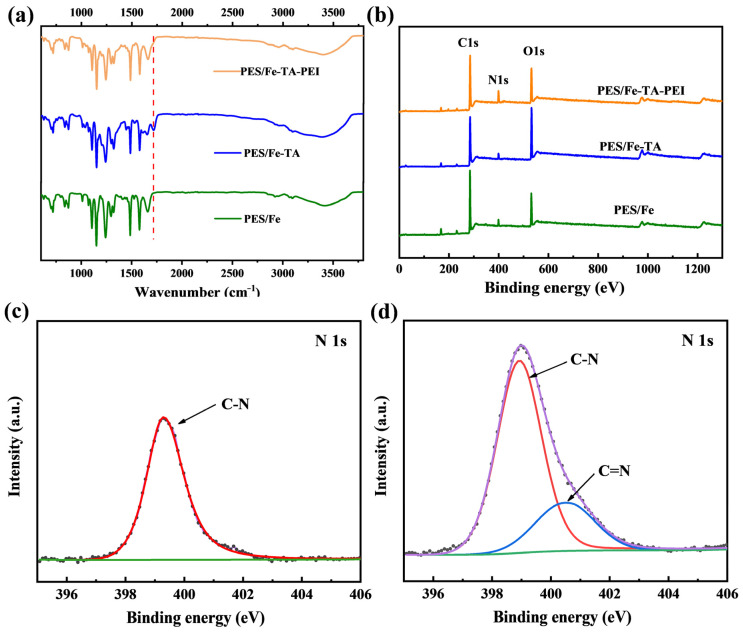
(**a**) ATR-FTIR spectra; (**b**) XPS spectra of the PES/Fe, PES/Fe-TA, and PES/Fe-TA-PEI membranes; High-resolution XPS spectra of N 1s (**c**) PES/Fe-TA membrane and (**d**) PES/Fe-TA-PEI membrane.

The LNMs were fabricated by coupling technique with NIPs method and grafting strategy. The surface photographs of each membrane are shown in Figure 1b. It can be found that the PES/Fe membrane exhibits yellow, while the color changed to yellow-brown after the TA coordination assembly modification. The pure PES membrane exhibits a white color, which has been reported in the previous work [27]. With the PES/Fe-TA membrane immersed in the PEI solution, the yellow-brown on the surface of the membrane becomes deeper. The color changes of PES/Fe, PES/Fe-TA, and PES/Fe-TA-PEI membranes indicate the successful introduction of TA and PEI. Figure 2a shows the FTIR spectra of the PES/Fe, PES/Fe-TA, and PES/Fe-TA-PEI, respectively. It can be seen that a new stretching vibration peak at 1717 cm^−1^ appears in the PES/Fe-TA membrane, which was assigned to the -C=O- bonds in TA [34]. After the introduction of PEI, this characteristic peak disappeared, which was assigned to the crosslinking reaction between TA and PEI. In addition, XPS analysis was employed to further confirm the surface element composition of the membranes and the result is shown in Figure 2b. It can be seen that the peak shapes of all the samples with respect to the C, N, and O elements are similar. N element appeared on the surface of PES/Fe and PES/Fe-TA membrane, which may be ascribed to the residues of PVP additive. The higher N 1s peak exhibited on the PES/Fe-TA-PEI membrane is evidence of the amine group from the PEI. To further confirmed the introduction of PEI, the N 1s peaks of the PES/Fe-TA membrane and PES/Fe-TA-PEI membrane were analyzed in detail. The PES/Fe-TA membrane showed a peak at 399.3 attributing to the C-N (Figure 2c) [35]. The N 1s core-level spectrum of PES/Fe-TA-PEI membrane results in two peaks at 399.3 and 400.5 eV, which were assigned to C-N and C=N, respectively (Figure 2d) [36]. The appearance of the C=N component confirmed the formation of a Schiff base reaction between TA and PEI. The quantitative analyses were made on the change of the element content of the three membranes (Table 1). Comparing to PES/Fe membrane, O element content on the surface of the assembled PES/Fe-TA nanofiltration membrane increased, which attributed to a large number of oxygen-containing functional groups of TA. After PEI grafting, N element content increased from 3.41% of PES/Fe-TA membrane to 8.86% of PES/Fe-TA-PEI nanofiltration, suggesting that PEI was successfully anchored onto the PES/Fe-TA layer. Overall, these results suggest that TA and PEI were successfully introduced onto the PES/Fe membrane.

**Figure 3:**
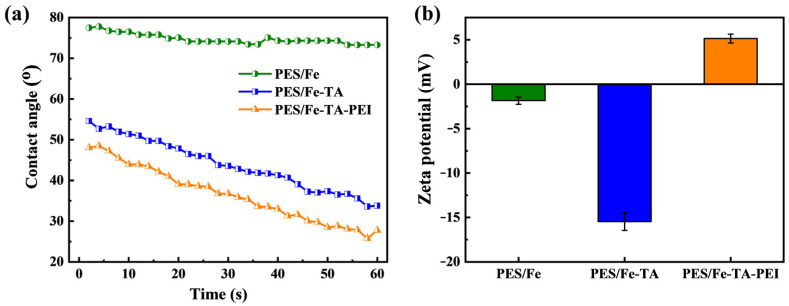
(**a**) WCA and (**b**) Zeta potentials (pH = 6.7) of the PES/Fe, PES/Fe-TA, and PES/Fe-TA-PEI membranes.

The wettability of the fabricated membranes was examined by the time-dependent WCA. As shown in Figure 3a, the WCA value of the PES/Fe membrane maintained smooth within 60 s and remained at about 75.8°. While the WCA value was only 54.6° for the PES/Fe-TA membrane and 46.5° for the PES/Fe-TA-PEI membrane, indicating that the enhanced hydrophilicity of the membrane. The WCA value of PES/Fe-TA membrane decreased from 54.6° to 33.8° within 60 s. This phenomenon was attributed to the introduction of phenolic hydroxyl group of TA on the membrane surface. With the PEI further cross-linking reaction, the contact angle decreases and drops to 43.2° within 10 s. This is due to the massive amidogen groups in the PEI and the phenolic hydroxyl groups in the unreacted TA, which increased the surface hydrophilicity of the membrane. Figure 3b shows the surface zeta potential measured at pH = 6.7. We can observe that PES/Fe and PES/Fe-TA membranes were negatively charged, while PES/Fe-TA-PEI membrane was positively charged. Compared with the PES/Fe membrane, the PES/Fe-TA membrane surface had more negative charge. This is because the introduction of more TA brought more anionic hydroxyl groups [33]. After introducing PEI on the surface of the PES/Fe-TA membrane, the chargeability of the membrane surface changed, and the Zeta potential changed from −15.46 mV to 5.14 mV. This is because a large number of amidogen groups in the PEI chain are protonated and cross-linked with the hydroxyl groups in TA and increase the positive charge of the PES/Fe-TA-PEI membrane surface. This phenomenon has been reported in other reports [3,37].

### 3.2. Effect of PEI Concentration on the Membrane Properties

**Figure 4:**
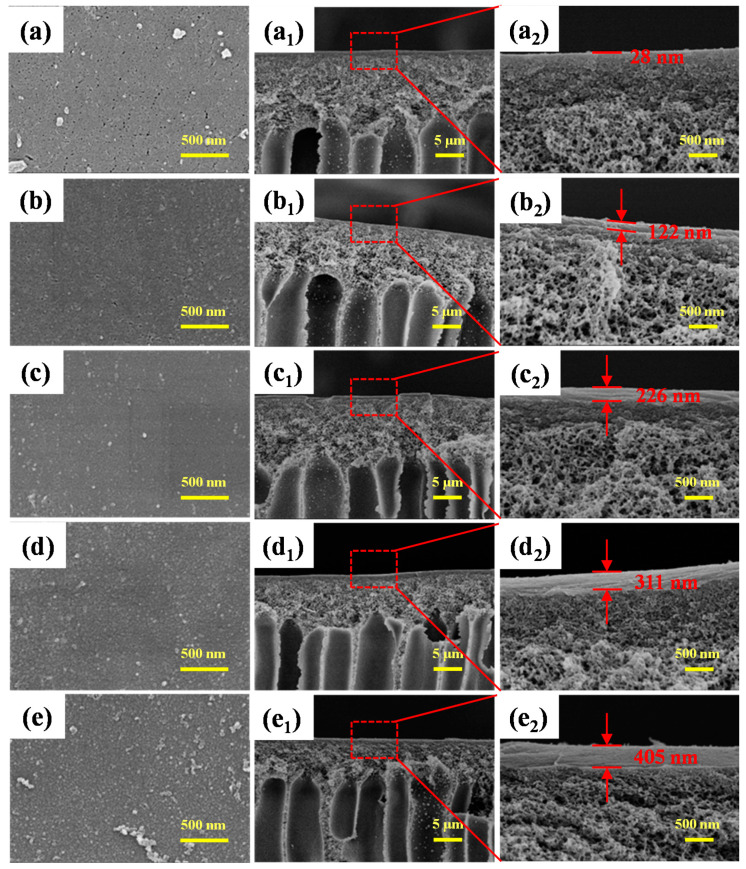
SEM images of the top surface and cross-section morphologies of PES/ Fe-TA-PEI membranes with different PEI concentrations ((**a**–**a_2_**) 0, (**b**–**b_2_**) 0.5, (**c**–**c_2_**) 1.0, (**d**–**d_2_**) 1.5, and (**e**–**e_2_**) 2.0 g/L).

Figure 4 shows SEM images of the top surface and cross-sectional morphology of PES/Fe-TA-PEI membranes cross-linked with different PEI concentrations (0, 0.5, 1.0, 1.5, and 2.0 g/L). It can be seen that the pores on the membrane surface gradually decreased and almost disappeared with the increase of PEI cross-linking concentration (Figure 4a–e). The membrane surface became rough and dense. This contributed to the formation of a new separation layer on the membrane surface. The corresponding cross-sectional images show the existence of double layers on supporting layer (Figure 4(a_1_–e_1_)). Thickness of the cross-linked separation layer on the membrane surface gradually increased with the increase of PEI concentration, and ranged from about 122 to 405 nm. This shows that PEI was successfully cross-linked on the surface of the membrane to form a dense active layer and the filtration performance was altered.

**Figure 5:**
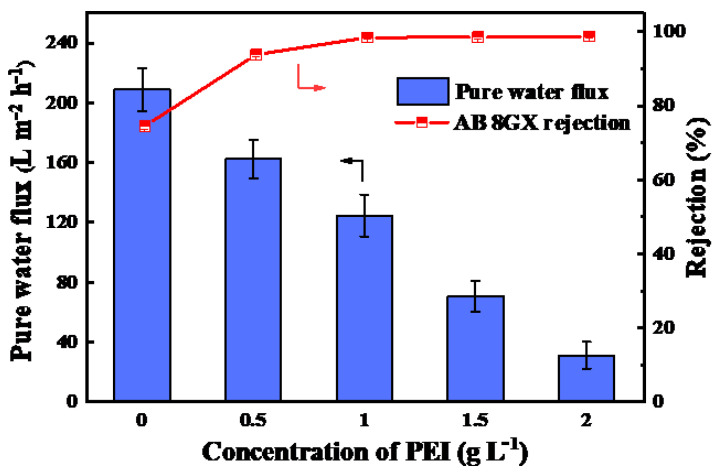
The pure water flux and AB 8GX rejection of PES/Fe-TA-PEI membranes with different PEI concentration.

The influences of PEI concentration on the pure water flux and AB 8GX rejection were examined under 2 bar and the results are shown in Figure 5. The pure water flux continuously decreased from 208.7 L·m^−2^·h^−1^ to 31.1 L·m^−2^·h^−1^ with increase of PEI concentration. The decrease of water flux for PES/Fe-TA-PEI membranes can be attributed that a new separation layer was formed on the membrane surface, which has been confirmed in Figure 4. Besides this, the rejection rate of AB 8GX elevated from 74.6% to 98.7%. The denser and positive charge of the selective layer were enhanced with increase of PEI concentration, which resulted in a high AB 8GX rejection for the PES/Fe-TA-PEI membranes with stronger electrostatic repulsive and aperture sieve. Comprehensively considering the water flux and dye rejection, the content of PEI was fixed at 1.0 g/L in the following experiments. The pure water permeability of nanofiltration membrane prepared under this condition was 62.3 L·m^−2^·h^−1^ bar^−1^, and the rejection rate of AB 8GX was 98.4%.

### 3.3. Filtration Performance

#### 3.3.1. Single Component Solution Filtration

**Figure 6:**
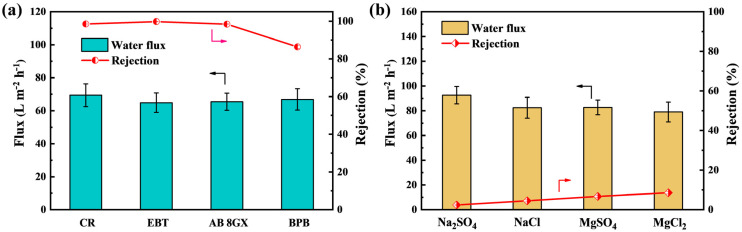
Separation performance of PES/Fe-TA-PEI membranes for different dye (**a**) and salt (**b**) solutions.

To further explore the NF separation performance, CR, EBT, AB 8GX, and BPB were chosen to evaluate the rejections of dyes with different charges and molecular size, and the results are shown in Figure 6a. The negatively charged dyes of CR and EBT have high rejection rates (>98%). On the one hand, due to the size exclusion effect, it was trapped in the pores of the separation layer. On the other hand, anionic dye molecules were adsorbed on the positive membrane surface and gradually formed a filter cake layer, which increased the rejection rate. Due to size exclusion and Donnan effects, the rejection rates of AB 8GX and BPB were 98.4% and 86.4%, respectively. It was worth noting that the rejection rate of AB 8GX was higher than that of BPB. The reason was that the result of the molecular weight of AB 8GX (1289 g/mol) was greater than that of BPB (670 g/mol). It showed that the sieving effect played a key role on the filtration of AB 8GX. At the same time, all permeation rates maintained a high level, and permeation flux of AB 8GX with the highest molecular weight still reached 65.7 L·m^−2^·h^−1^. As shown in Figure 6b, the rejection ratios of the PES/Fe-TA-PEI membranes for several inorganic salts, Na_2_SO_4_, NaCl, MgSO_4_, and MgCl_2_, were all <10%. This indicates that the PES/Fe-TA-PEI membranes exhibited efficient dye and salt selective separation. Notably, the PES/Fe-TA-PEI membranes exhibited a salt rejection sequence of MgCl_2_ > MgSO_4_ > NaCl > Na_2_SO_4_, which conformed the typical positively charged NF membranes. This could be explained by the electrostatic interaction and molecular sieving effects [38]. The electrostatic attraction of the positively charged membrane surface to divalent anion (SO_4_^2−^) is stronger than that of the monovalent anion (Cl^−^), resulting in sulfate passing through the membrane more easily. In addition, the Na^+^ with the hydrated radius of 0.358 nm had a faster permeation rate than the Mg^2+^ (0.428 nm) due to the sieving effect. Besides this, compared to monovalent cations (Na^+^), divalent cations (Mg^2+^) had stronger repulsive interactions with the positively charged membrane surface. Thus, the membrane exhibited the highest rejection for MgCl_2_ (8.6%) and the lowest rejection for Na_2_SO_4_ (2.4%). The above results demonstrate that the prepared PES/Fe-TA-PEI membranes have potential applications in dye/salt separation.

#### 3.3.2. Separation Performance to Dye/Salt

**Figure 7:**
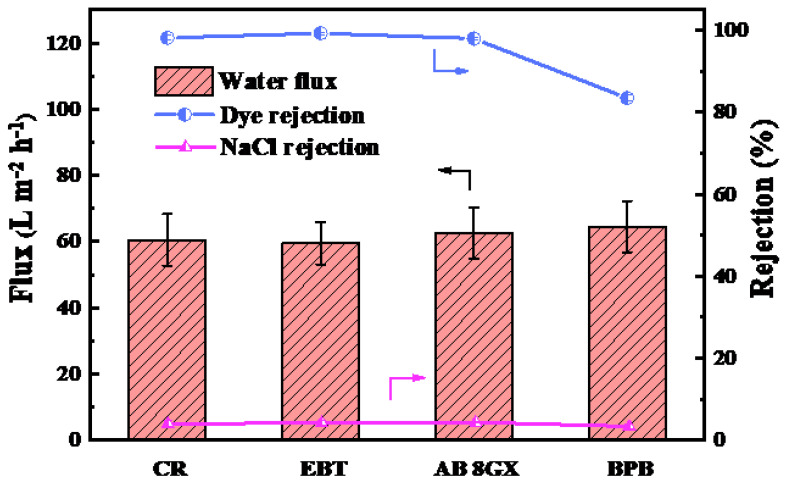
Separation performance of dye/salt composite system.

The separation performance of the dye/salt mixed system was investigated through different dye and NaCl solutions. As shown in Figure 7, the permeation flux and dye rejection rate of the PES/Fe-TA-PEI membrane were 59.5~64.5 L·m^−2^·h^−1^ and 83.5~99.3%, respectively, which was slightly lower than those of single system. This was because salt enhanced the dispersibility for dye molecules in the solution, and uniformly dispersed dye molecules were easily adsorbed on membrane pores and pass-through membrane, thereby reducing filtration performance of PES/Fe-TA-PEI membrane [3]. Although the dye rejection rates of PES/Fe-TA-PEI membranes were slightly lower than that of a single system, the rejection rates of anionic dyes remained above 98% owing to the dual effects of pore size sieving and dye adsorption on the membrane surface. The cationic retention of dyes AB 8GX (98.0%) and BPB (83.5%) was mainly by virtue of the electrostatic repulsion and size sieving effects. In addition, it can be seen from the figure that the NaCl rejection rate in the cationic dye/salt mixed system (3.15~4.15%) was slightly higher than that in the anionic dye/salt mixed system (2.9~4%). This was because the cationic dye molecules adsorbed on the membrane surface increase the positive charge of the membrane surface, thereby promoting the retention of NaCl. Moreover, compared with a single NaCl solution system (NaCl rejection rate of 4.5%), the PES/Fe-TA-PEI membrane has a reduced NaCl rejection rate (2.9~4.15%) in the dye salt mixed system. This shows that the PES/Fe-TA-PEI membrane has great potential and application prospects in dye desalination.

### 3.4. Antifouling Properties and Long-Term Stability

**Figure 8:**
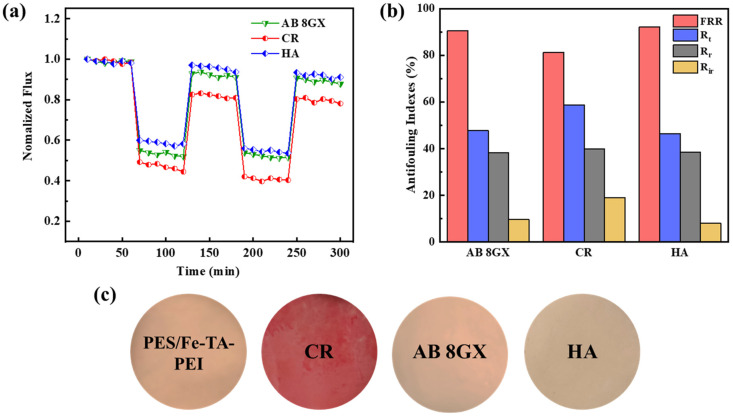
Membrane fouling during filtration of CR, AB 8GX, and HA solution with PES/Fe-TA-PEI membranes: (**a**) normalized flux, (**b**) antifouling indexes, and (**c**) membrane comparative photographic images before and after fouling.

Furthermore, we evaluated the antifouling performance and structural stability of the PES/Fe-TA-PEI LNM, which are important issues for LNMs in practical operation. In order to evaluate the antifouling performance of the PES/ Fe-TA-PEI membrane, the negatively charged CR and positively charged AB 8GX were used as model pollutants to test the time-dependent water fluxes. As shown in Figure 8a, the normalized flux of membranes had sharply declined when the dye solution is filtered, and the membrane suffered from different degrees of contamination. Subsequently, the normalized flux recovered to different levels and then remained stable after 30 min washing. In addition, the antifouling indexes of the PES/Fe-TA-PEI membrane for CR and AB 8GX were calculated and presented in Figure 8b. After two cycles, the FRR to CR and AB 8GX were 81.1% and 90.5%, and the corresponding Rt were 58.7% and 47.7%, respectively. The anti-fouling ability of positively charged AB 8GX was higher than that of negatively charged CR. This is because the latter was more easily adsorbed on the surface of the positively charged membrane through electrostatic action to form a densely packed fouling layer (Figure 8c). Furthermore, the antifouling measurements of the PES/Fe-TA-PEI membrane were conducted using HA as a representative pollutant of natural organic matter. The normalized water flux of the membrane was recovered to 92.2% after two cycles. Although HA was negatively charged, giving rise to electrostatic interaction between HA and membrane surface in the filtration of HA solution, the large molecular size and hydrophilic membrane surface restrict its penetration into membrane matrix. The fouling deposits could be taken away by hydraulic cleaning. In general, the hydrophilic PES/Fe-TA-PEI membrane exhibited good antifouling performance.

**Figure 9:**
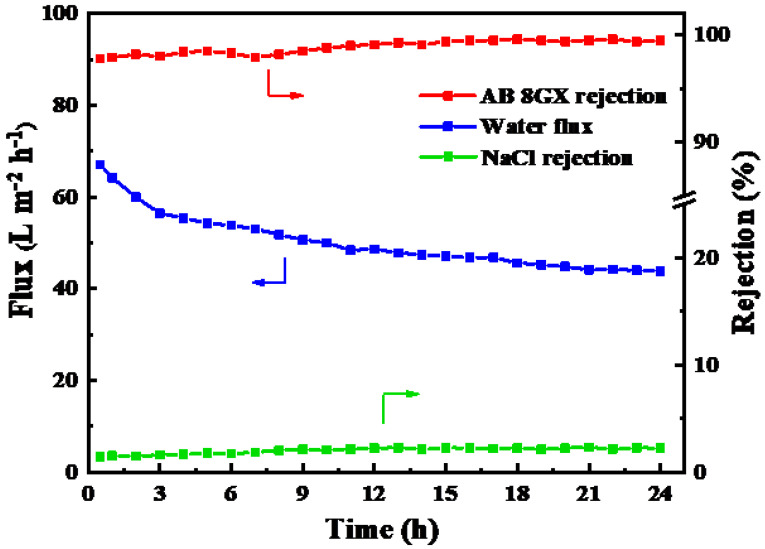
The long-time operation stability of PES/Fe-TA-PEI membranes for AB 8GX/NaCl mixture solution (feed: 0.1 g/L AB 8GX and 1 g/L NaCl).

In order to further explore the structure and separation stability of the PES/Fe-TA-PEI membrane, a mixture solution of 0.1 g/L AB 8GX and 1 g/L NaCl was used as the feed solution for a 24-hour filtration test. The results are shown in Figure 9. It can be seen that the permeation flux of the membrane decreased slightly in the initial stage, which was attributed to the deposition of dye molecules on the membrane surface. However, the membrane showed a stable permeation flux as high as 45.2 L·m^−2^·h^−1^ when the adsorption reached dynamic equilibrium. Additionally, the rejection rate of membrane toward AB 8GX and NaCl was relatively stable during the whole filtration process. The rejection rate of AB 8GX was greater than 98.4% and the rejection rate of NaCl was less than 5.3%. These results indicate that the prepared membrane possesses stable structure and filtration performance during the filtration process of dye/salt solution, which further demonstrates its practical application potential in dyeing wastewater treatment.

### 3.5. Performance Comparison with Other Membranes Reported in the Literature

The separation performance of PES/Fe-TA-PEI membrane obtained in this work was compared with other loose nanofiltration membranes. Table 2 lists the permeability, dye rejection, and salt rejection of different membranes [13,21,39,40,41,42,43,44]. It can be seen that the PES/Fe-TA-PEI membrane prepared in this study showed relatively high permeate flux and high rejection for dyes as well as low salts rejections in comparison with the listed membranes. After comprehensive comparison, our work exhibited superior performance and potential in the practical application of dye/salt separation.

## 4. Conclusions

In this work, a novel composite loose NF membrane with high permeate flux and good dye/salt separation performance was fabricated through a facile and scalable method. Firstly, the PES/Fe-TA nanofiltration membrane was prepared by the coordination reaction between TA and Fe^3+^. Then, the PEI was grafted on the surface of the membrane by crosslinking reaction with TA. The optimized PES/Fe-TA-PEI membrane achieved the permeation flux of 69.4, 64.8, 65.7 and 66.9 L·m^−2^·h^−1^ to CR, EBT, AB 8GX and BPB aqueous solutions, with the corresponding rejection of 98.5%, 99.8%, 98.4%, 86.4%, respectively. The retention for the inorganic salts was 8.6% for MgCl_2_, 6.7% for MgSO_4_, 4.5% for NaCl, and 2.4% for Na_2_SO_4_. In addition, the membrane also showed a stable and high dye rejection rate (>98.4%) and low salt permeability (<5.3%) in the 24-h test. Furthermore, the membrane presented an excellent antifouling performance. Thus, all these results suggest that our developed PES/Fe-TA-PEI membrane had a great potential application in dye/salt fractionation for saline textile wastewater treatment.

## Figures and Tables

**Table 1:** The element content of various membranes.

Samples	C (%)	N (%)	O (%)	O/N
PES/Fe	77.65	3.90	18.45	4.73
PES/Fe-TA	68.24	3.41	28.34	8.31
PES/Fe-TA-PEI	70.65	8.86	20.49	2.31

**Table 2:** Comparison of the performance of the NF membranes in the literatures.

Membranes	Permeability (LMH/bar)	Types of Dye	Dye Rejection (%)	Salt Rejection (%)	Refs.
CA NF	8.1	Reactive Orange 12 Reactive Black 5	99.9 99.0	10 (NaCl) 40 (Na_2_SO_4_)	[39]
Sepro NF 2A	10.1	Direct red	99.9	21.2 (NaCl)	[40]
Sepro NF 6	13.7	Direct red 80 Congo red	>99.6 >99.6	<33.3 (NaCl)	[13]
TA/GOQDs-1	11.7	Congo red Methyl blue	99.8 97.6	17.2 (NaCl) 66.7 Na_2_SO_4_)	[41]
PAN-PEI-GA	25.5	Congo red Methyl Blue	97.1 97.3	5.0 (NaCl)	[21]
PAN-DR80	28.4	Congo red	99.8	12.4 (NaCl)	[42]
CaCO_3_/PEI-GA	48.5	Congo red Methyl blue	99.6 97.7	6.9 (NaCl) 10.2 Na_2_SO_4_)	[43]
LNFM-2	53.2	Congo red Direct red 23 Reactive Blue 2	99.6 95.2 99.6	5.6 (NaCl)11.0 Na_2_SO_4_)	[44]
PES/Fe-TA-PEI	62.3	Congo red Eriochrome black T	98.5 99.8	4.5 (NaCl) 8.6 (MgCl_2_)	This work
		Alcian blue 8GX	98.4	2.4 (Na_2_SO_4_)	

## Data Availability

This study did not report any data in public datasets analyzed or generated.
